# Collapse Pressure Analysis of Transversely Isotropic Thick-Walled Cylinder Using Lebesgue Strain Measure and Transition Theory

**DOI:** 10.1155/2014/240954

**Published:** 2014-01-09

**Authors:** A. K. Aggarwal, Richa Sharma, Sanjeev Sharma

**Affiliations:** Department of Mathematics, Jaypee Institute of Information Technology, A-10, Sector 62, Noida 201307, India

## Abstract

The objective of this paper is to provide guidance for the design of the thick-walled cylinder made up of transversely isotropic material so that collapse of cylinder due to influence of internal and external pressure can be avoided. The concept of transition theory based on Lebesgue strain measure has been used to simplify the constitutive equations. Results have been analyzed theoretically and discussed numerically. From this analysis, it has been concluded that, under the influence of internal and external pressure, circular cylinder made up of transversely isotropic material (beryl) is on the safer side of the design as compared to the cylinders made up of isotropic material (steel). This is because of the reason that percentage increase in effective pressure required for initial yielding to become fully plastic is high for beryl as compared to steel which leads to the idea of “stress saving” that reduces the possibility of collapse of thick-walled cylinder due to internal and external pressure.

## 1. Introduction

Lame in 1852 first studied the hollow circular cylinder under pressure which is widely used in structures, aerospace, and nuclear reactors. The problems of homogeneous and isotropic circular cylinder under internal pressure have been found in most of the standard elasticity and plasticity books [1, 2]. These days, pressurized (internal and external) cylinders have become a point of interest of researchers due to their wide application in nuclear industry, especially in advanced small and medium-sized light water reactors. A steam generator tube is an example of the problem of circular cylinder under internal and external pressure, in which primary coolant flows outside the tubes (external pressure), while secondary water flows inside the tubes (internal pressure). Another example is pipelines under seawater to transport gas, oil, and so forth. In general, vessels under high pressure require a strict analysis for an optimum design for reliable and secure operational performance and thus efforts were continually made to increase reliability. Solutions are obtained either analytically or with the implementations of numerical methods. Rimrott [3] used the assumptions of constant density, zero axial strain, and distortion-energy law to calculate creep strain rate and stresses in a thick-walled closed-end hollow cylinder under internal pressure made of an isotropic and homogeneous material under internal pressure. A known creep strain rate versus stress relation is then used to solve this specific problem. Zhao et al. [4] discussed elastic-plastic analysis of a thick-walled cylinder under internal pressure. They involve two parametric functions and piecewise linearization of the stress-strain curve. A deformation type of relationship is combined with Hooke's law in such a way that stress-strain law has the same form in all linear segments, but each segment involves different material parameters. This technique involves the use of deformed geometry to satisfy the boundary and other relevant conditions. Yoo et al. [5] investigated the collapse pressure of cylinders with intermediate thickness subjected to external pressure based on finite element (FE) analysis. According to the concept of the partial safety factor, the yield strength was concluded to be the most sensitive, and the initial ovality of tube was not so effective in the proposed collapse pressure estimation model. Perry and Aboudi [6] discussed the optimal design of a modern gun barrel with two main objectives: the first one is to increase its strength-weight ratio and the second is to extend its fatigue life. This can be carried out by generating a residual stress field in the barrel wall. A Von-Mises' yield criterion, isotropic strain hardening with the Prandtl-Reuss theory has been taken into the consideration with Bauschinger effect and plane stress conditions. The stresses are calculated incrementally by using the finite difference method. Davidson et al. [7] determine the residual-stress distribution as a function of magnitude of overstrains and diametric ratio and discuss the effects on the reyielding characteristics of cylinders. All these authors considered yield criterion, jump conditions, and linear strain measure to calculate the stresses using the concept of infinitesimal strain theory. According to the approach of the above authors, the spectrum of deformations is divided into two regions; that is, one is elastic region and another one is plastic region which is physically not possible because transition from one state into another state is a continuous phenomenon. At transition, the fundamental structure undergoes a change and the particles constituting the material rearrange themselves and give rise to spin, rotation, vorticity, and other nonlinear effects. This suggests that at transition, nonlinear terms are very important and ignoring them may not represent the real physical phenomenon.

Transition theory [8, 9] does not require any of the above assumptions and thus solves a more general problem using the concept of generalized strain measure [10], which not only gives the well-known strain measures but can also be used to find the stresses in plasticity and creep problems by determining the asymptotic solution at the transition points of the governing differential equations. This theory has been applied to many problems; for example, Sharma et al. [11] analyzed thermal elastic-plastic stresses in transversely isotropic thick-walled rotating cylinder under internal pressure. Aggarwal et al. [12] investigated safety factors in thick-walled functionally graded cylinder under internal and external pressure and concluded that functionally graded cylinder is a better choice for designers as compared to cylinders made up of homogeneous materials. Aggarwal et al. [13] discussed safety factors in creep nonhomogeneous thick-walled circular cylinder under internal and external pressure with steady state temperature and concluded that cylinder made up of nonhomogeneous material is better choice for designing as compared to cylinder made up of homogeneous material.

If a continuous phenomenon is represented by a spectrum, nonlinearity exhibits itself at its ends, which corresponds to transition state. Thus the classical measures of deformation are inadequate to deal with such transitions. In classical theory, such transition state requires semiempirical laws to match the solutions and thus discontinuities have to be introduced, where they do not exists. A continuum approach necessarily means the introduction of nonlinear measures. If in a very small interval the number of fluctuations is very large, the concept of ordinary measure based on Riemann integral fails and Lebesgue measures may be used. In like manner, the generalized measures given by weighted integral representations give very satisfactory results in problems of plasticity and creep. Seth has defined the generalized principal strain measure *ε*_*ii*_ by taking the Lebesgue integral of the weighted function

(1) εii=∫0eiiA[(1−2eiiA)](n/2)−1deiiA=  1n[1−(1−2eiiA)(n/2)−1],

where *n* is the measure and  *e*_*ii*_^*A*^  is the principal Almansi strain component. For uniaxial case,

(2) $$\begin{array}{c} e={\left[\frac{1}{n}\left\{1-{\left(\frac{l_{0}}{l}\right)}^{n}\right\}\right]}^{m}, \end{array}$$

where *m* is the irreversibility index and *l*_0_ and *l* are the undeformed and deformed lengths of the rod, respectively. For *n* = −2, −1, 0, 1, 2 in (2) gives the Green, Hencky, Swainger, and Almansi measure, respectively, where in all cases *m* = 1. The most important contribution made by the generalized Lebesgue measure is that they eliminate the use of empirical laws and jump conditions.

As we know the permissible stress of any material is some proportion of the yield or ultimate stress of the material and, as such, it incorporates a “safety factor.” This safety factor provides a margin against the collapse condition of the cylinder that occurs due to high pressure. Since the condition of high pressure can cause failure of the cylinder, therefore it is necessary to examine the state of the cylinder at collapse and to design the cylinder accordingly. In order to provide guidance on a design and integrity evaluation of a cylinder under pressure, the failure characteristics of a cylinder should be considered carefully. In this paper, it is our main aim to eliminate the need of assuming semiempirical laws, yield condition, creep-strain laws, jump conditions, and so forth to obtain the collapse pressure in transversely isotropic thick-walled circular cylinder under internal and external pressure using generalized Lebesgue strain measure. The stresses are calculated in both transition and fully plastic state.

## 2. Mathematical Formulation of Problem

**Figure 1:**
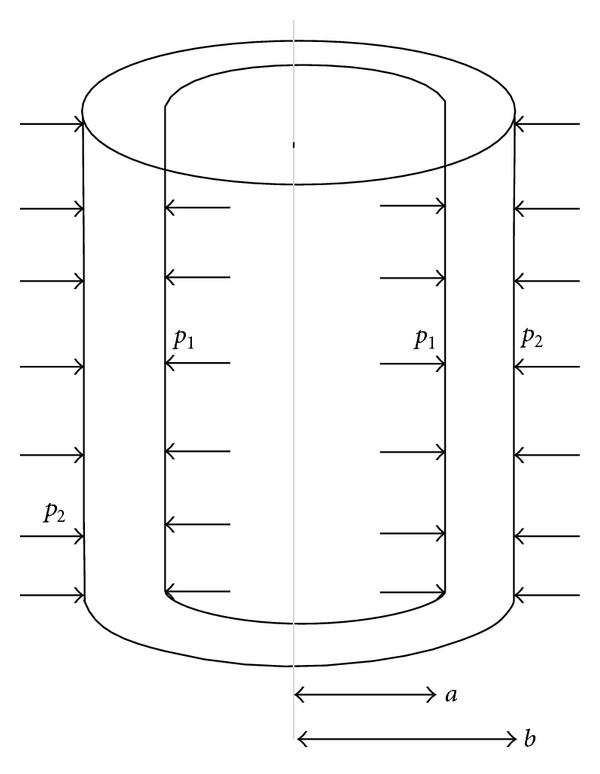
Geometry of thick-walled transversely isotropic cylinder under internal and external pressure.

We consider a thick-walled circular cylinder made up of transversely isotropic material with internal and external radii “*a*” and “*b,*” respectively, subjected to internal pressure *p*_1_ and external pressure *p*_2_ as shown in the Figure 1. The cylinder is taken so large that the plane sections remain plane during the expansion, and hence the longitudinal strain is the same for all elements at each stage of the expansion.

The components of the displacement in cylindrical polar coordinates are given by

(3) $$\begin{array}{c} u=r\left(1-\beta \right),\;v=0,\;\;w=dz, \end{array}$$

where *β* is a function of $r=\sqrt{x^{2}+y^{2}}$ only and *d* is a constant.

The generalized components of strain [10] are given as follows:

(4) err=1n[1−(rβ′+β)n]  ,    eθθ=1n[1−βn],ezz=1n[1−(1−d)n],    erθ=eθz=ezr=0.

The stress-strain relations for transversely isotropic material are

(5) $$\begin{array}{c} T_{rr}=C_{11}e_{rr}+\left(C_{11}-2C_{66}\right)e_{\theta \theta }+C_{13}e_{zz},\\ T_{\theta \theta }=\left(C_{11}-2C_{66}\right)e_{rr}+C_{11}e_{\theta \theta }+C_{13}e_{zz},\\ T_{zz}=C_{13}e_{rr}+C_{13}e_{\theta \theta }+C_{33}e_{zz},\\ T_{zr}=T_{\theta z}=T_{r\theta }=0. \end{array}$$

Using (4) in (5), we get

(6) Trr=(C11n)[1−(β+rβ′)n] +[(C11−2C66)n][1−βn]+C13ezz,Tθθ=[(C11−2C66)n][1−(β+rβ′)n] +(C11n)[1−βn]+C13ezz,Tzz=(C13n)[1−(β+rβ′)n] +(C13n)[1−βn]+C33ezz,Trθ=Tθz=Trz=0.

Equations of equilibrium are all satisfied except

(7) $$\begin{array}{c} \frac{d}{dr}\left(T_{rr}\right)+\left(\frac{T_{rr}-T_{\theta \theta }}{r}\right)=0. \end{array}$$

## 3. Identification of Transition Point

We know that as the point in the material has yielded, the material at the neighbouring points is on its way to yield rather than remain in its complete elastic state or fully plastic state. Thus we can assume that there exists some state in-between elastic and plastic which is called transition state. So, at transition the differential system defining the elastic state should attain some criticality. The differential equation which comes out to be nonlinear at transition state is obtained by substituting equations (6) in (7):

(8) nPC11βn+1(1+P)n−1dPdβ=−nPC11βn(1+P)n −(C11−2C66)nPβn +2C66[1−βn(1+P)n] −2C66(1−βn),

where *rβ*′ = *βP*.

The critical points or transitional points of (8) are *P* → −1 and *P* → ±*∞*.

The boundary conditions are given by

(9) $$\begin{array}{c} T_{rr}=-p_{1}\;\text{at}\;r=a,\\ T_{rr}=-p_{2}\;\text{at}\;r=b. \end{array}$$

The resultant axial force is given by

(10) $$\begin{array}{c} 2\pi \int_{a}^{b}rT_{zz}dr=\pi a^{2}\left(p_{1}-p_{2}\right). \end{array}$$

## 4. Mathematical Approach

The material from elastic state goes into plastic state as *P* → ±*∞* or to creep state as *P* → −1 under internal and external pressure. It has been shown [8, 9, 11–13] that the asymptotic solution through the principal stress leads from elastic to plastic state at the transition point *P* → ±*∞*. For finding the plastic stress at the transition point *P* → ±*∞*, we define the transition function *R* in terms of *T*_*rr*_ as

(11) R=2(C11−C66)+nC13ezz−nTrr=βn[C11−2C66+C11(1+P)n].

Taking the logarithmic differentiation of (11) with respect to “*r*,” with asymptotic value as *P* → ±*∞*, on integration yields

(12) $$\begin{array}{c} R=A_{1}r^{-C_{1}}, \end{array}$$

where *A*_1_ is a constant of integration and *C*_1_ = 2*C*_66_/*C*_11_.

Using (11) and (12), we get

(13) $$\begin{array}{c} T_{rr}=C_{3}-\left(\frac{A_{1}}{n}\right)r^{-C_{1}}. \end{array}$$

Using boundary condition (9) in the above equation, we get

(14) $$\begin{array}{c} A_{1}=nb^{C_{1}}\left[\frac{p}{{\left(b/a\right)}^{C_{1}}-1}\right],\;\;C_{3}=\left[\frac{p}{{\left(b/a\right)}^{C_{1}}-1}\right]. \end{array}$$

Substituting the value of *A*_1_ and *C*_3_ in (13), we get

(15) $$\begin{array}{c} T_{rr}=\left[\frac{p_{1}-p_{2}}{{\left(b/a\right)}^{C_{1}}-1}\right]\left[1-{\left(\frac{b}{r}\right)}^{C_{1}}\right]-p_{2}. \end{array}$$

Using (15) in (7), we have

(16) $$\begin{array}{c} T_{\theta \theta }=\left[\frac{p_{1}-p_{2}}{{\left(b/a\right)}^{C_{1}}-1}\right]\left[1-\left(1-C_{1}\right){\left(\frac{b}{r}\right)}^{C_{1}}\right]-p_{2}, \end{array}$$

(17) Tzz=C132(C11−C66)[(p1−p2(b/a)C1−1)×(2−(2−C1)(br)C1)] +a2(p1−p2)b2−a2−a2C13(p1−p2)(C11−C66)(b2−a2).

From (15) and (16), we get

(18) $$\begin{array}{c} T_{\theta \theta }-T_{rr}=\left[\frac{p_{1}-p_{2}}{{\left(b/a\right)}^{C_{1}}-1}\right]C_{1}{\left(\frac{b}{r}\right)}^{C_{1}}. \end{array}$$

From the above equation it is found that the value of |*T*_*θθ*_ − *T*_*rr*_| is maximum at *r* = *a*, which means yielding of the cylinder will take place at the internal surface. Therefore, we have

(19) $$\begin{array}{c} {\left|T_{\theta \theta }-T_{rr}\right|}_{r=a}=\left|\left[\frac{p_{1}-p_{2}}{{\left(b/a\right)}^{C_{1}}-1}\right]C_{1}{\left(\frac{b}{a}\right)}^{C_{1}}\right|\equiv Y\left(\text{say}\right). \end{array}$$

Let *p*_1_ − *p*_2_ = *p*

The pressure required for initial yielding is given by

(20) $$\begin{array}{c} \left|P_{i}\right|=\left|\frac{p}{Y}\right|=\frac{\left[{\left(b/a\right)}^{C_{1}}-1\right]}{C_{1}{\left(b/a\right)}^{C_{1}}}, \end{array}$$

where (*p*_1_/*Y*)−(*p*_2_/*Y*) = *P*_*i*1_ − *P*_*i*2_ = *P*_*i*_.

Using (20) in (15), (16), and (17), we get transitional stresses as

(21) TrrY=[Pi(b/a)C1−1][1−(br)C1]−Pi2,TθθY=[Pi(b/a)C1−1][1−(1−C1)(br)C1]−Pi2,TzzY=C132(C11−C66)[(Pi(b/a)C1−1) ×(2−(2−C1)(br)C1)] +a2Pib2−a2−a2C13Pi(C11−C66)(b2−a2).

Equation (21) give elastic-plastic transitional stresses in thick-walled cylinder under internal and external pressure.

For fully plastic state (*C*_1_ → 0), (20) becomes

(22) $$\begin{array}{c} {\left|T_{\theta \theta }-T_{rr}\right|}_{r=b}=\left|\left\{\frac{p}{\log\left(b/a\right)}\right\}\right|\equiv Y_{1}\left(\text{say}\right), \end{array}$$

where *P*_*f*_ = (*p*_1_ − *p*_2_)/*Y*_1_ = *P*_*f*1_ − *P*_*f*2_.

The stresses for fully plastic state are

(23) TrrY1=log(rb)−Pf2,TθθY1=(1+log(rb))−Pf2,TzzY1=C132(C11−C66)(1+2log(rb)) +a2log(b/a)b2−a2−a2C13log(b/a)(C11−C66)(b2−a2).

Now we introduce the following nondimensional components as

(24) R=(rb),  R0=(ab),  σrt=[TrrY],σθt=[TθθY],  σzt=[TzzY],  σrf=[TrrY1],σθf=[TθθY1],  σzf=[TzzY1].

The necessary effective pressure required for initial yielding is given by (20) in nondimensional form as

(25) $$\begin{array}{c} \left|P_{i}\right|=\frac{\left[{\left(R_{0}\right)}^{C_{1}}-1\right]}{C_{1}{\left(R_{0}\right)}^{-C_{1}}}. \end{array}$$

The transitional stresses given by (21) become

(26) σrt=[Pi(R0)−C1−1][1−(R)−C1]−Pi2,σθt=[Pi(R0)−C1−1][1−(1−C1)(R)−C1]−Pi2,σzt=C132(C11−C66)[(Pi(R0)−C1−1)×(2−(2−C1)(R)−C1)] +Pi(R0−2−1)  −C13Pi(C11−C66)(R0−2−1).

The effective pressure required for full plasticity is given by

(27) $$\begin{array}{c} P_{f}=\log\left(\frac{1}{R_{0}}\right). \end{array}$$

Now stresses for full plasticity are obtained by taking *C*_1_ → 0; we have

(28) σrf=−(Pf1−Pf1)log(R)log(R0)−Pf2,σθf=−(Pf1−Pf2)[1+log(R)]log(R0)−Pf2,σzf=C132(C11−C66)(1+2log(R)) +log(R0)(1−R0−2)−C13log(R0)(C11−C66)(1−R0−2).

## 5. Numerical Discussion and Conclusion

**Figure 2:**
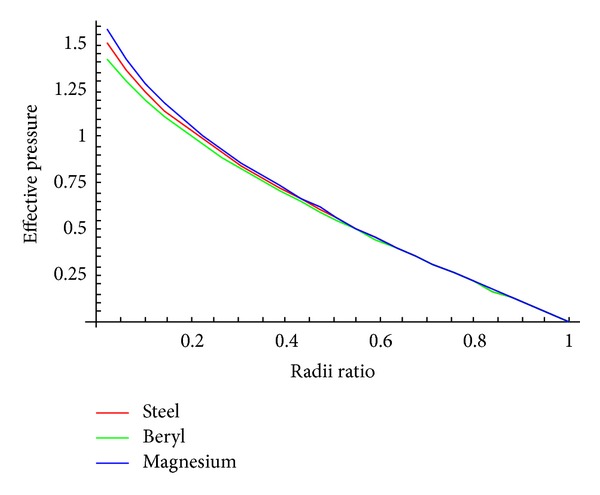
Effective pressure required for initial yielding for isotropic (seel) and transversely isotropic material (beryl and Steel).

**Figure 3:**
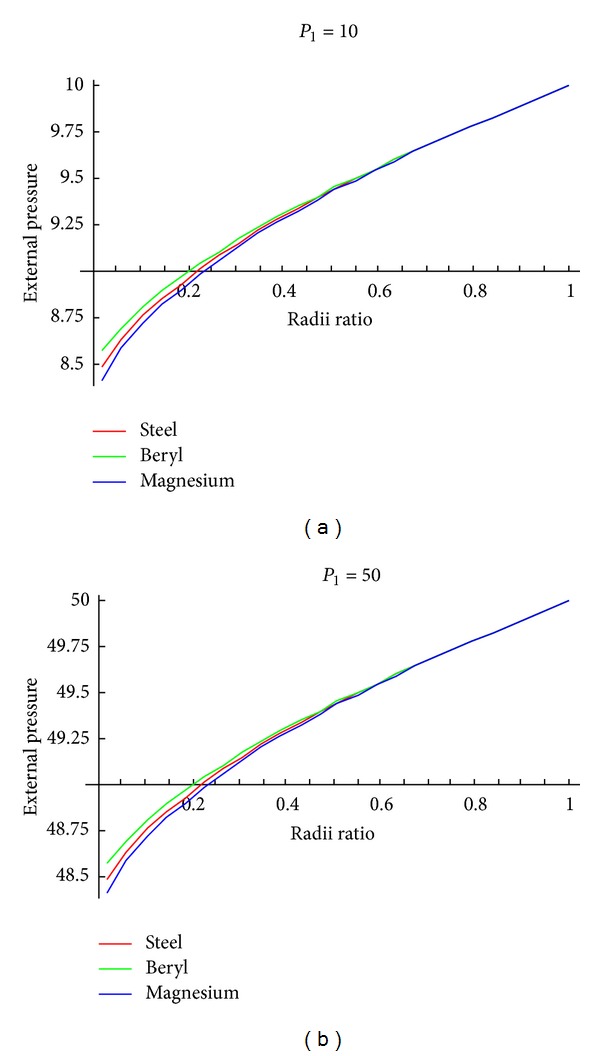
External pressure required for initial yielding when internal pressure *P*_1_ = 10, 50 for isotropic (steel) and transversely isotropic material (beryl and steel).

To observe the effect of pressure required for initial yielding and fully plastic state against various radii ratios, Tables 2–4 and Figures 2 and 3 using Table 1 have been drawn.

From Table 2, it can be seen that in case of isotropic material (steel) effective pressure required for initial yielding and fully plastic state is high for the cylinder whose radii ratio is 0.2, as compared to the cylinder with other radii ratios, that is, 0.3, 0.4, and so forth. It has also been noted that percentage increase in effective pressure required for initial yielding to become fully plastic is high for the cylinder with radii ratio 0.2 as compared to cylinder with other radii ratios. It has been noticed from Tables 3 and 4 that in case of transversely isotropic materials (beryl and magnesium) effective pressure required for initial yielding to fully plastic state is again high for cylinder with radii ratio 0.2 as compared to the cylinder with other radii ratios. It has also been observed from Tables 2–4 that percentage increase in effective pressure required for initial yielding to become fully plastic is high for cylinder made up of beryl material as compared to magnesium and steel material. It has also been observed from Table 2 that, for isotropic material (steel), external pressure required for initial yielding and fully plastic state when internal pressure is given (say, *P*_1_ = 10) is high for the cylinder with radii ratio, 0.5 as compared to cylinders with less radii ratio, while percentage increase in external pressure required for initial yielding to become fully plastic is high for the cylinder with lesser radii ratio as compared to cylinder with higher radii ratio. From Tables 3 and 4, it has been noticed that, for transversely isotropic material, external pressure required for initial yielding and fully plastic state is again high for cylinder with high radii ratio as compared to cylinder with less radii ratio while percentage increase in external pressure required for initial yielding to become fully plastic is high for cylinder with lesser radii ratio as compared to higher radii ratio cylinder. From Tables 2–4, it can be seen that, with the increase in internal pressure, external pressure required for initial yielding and fully plastic state also increases. Also, it has been noted that this percentage increase in external pressure required for initial yielding to become fully plastic is high for cylinder made up of beryl as compared to cylinder made up of steel and magnesium.

From Figure 2, it is noticed that effective pressure required for initial yielding is maximum at internal surface and this effective pressure is more for magnesium as compared to beryl as well as steel. From Figure 3, it can be seen that external pressure required for initial yielding for the cylinder with internal pressure (=10) is maximum at external surface. Also it has been observed that external pressure required for initial yielding is high for beryl as compared to steel and magnesium. As internal pressure increases, external pressure required for initial yielding also increases accordingly.

**Figure 4:**
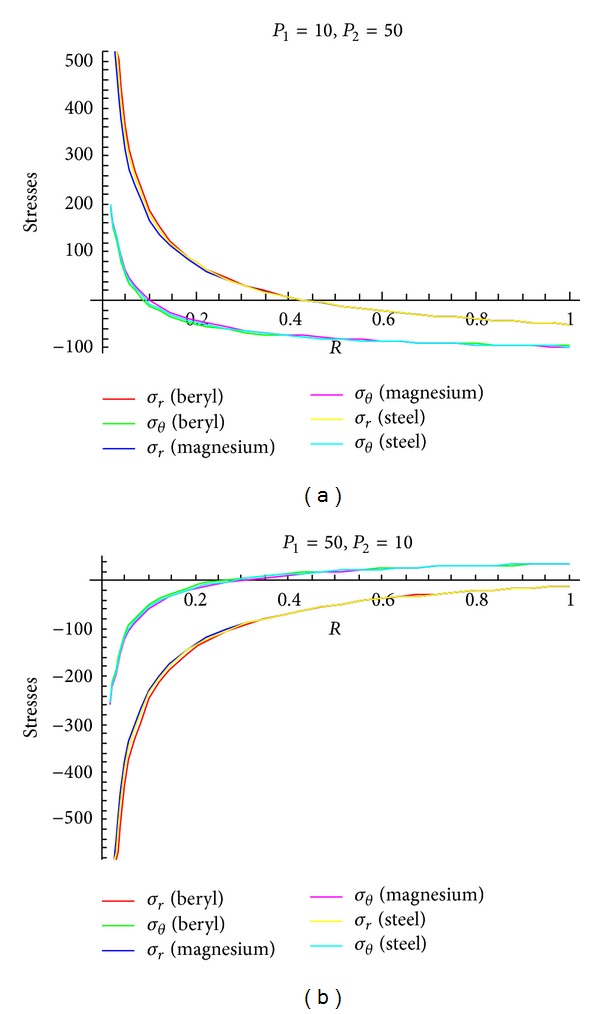
Transitional stresses when internal pressure = 10, external pressure = 50 and internal pressure = 50, external pressure = 10, respectively.

**Figure 5:**
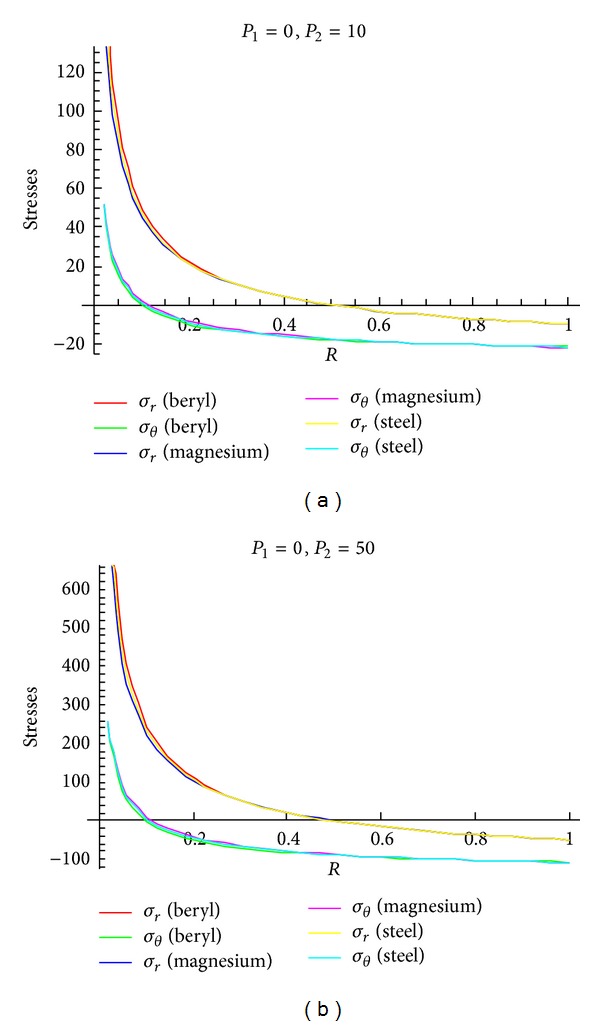
Transitional stresses when external pressure *P*_2_ = 10 and external pressure *P*_2_ = 50 (without internal pressure).

**Figure 6:**
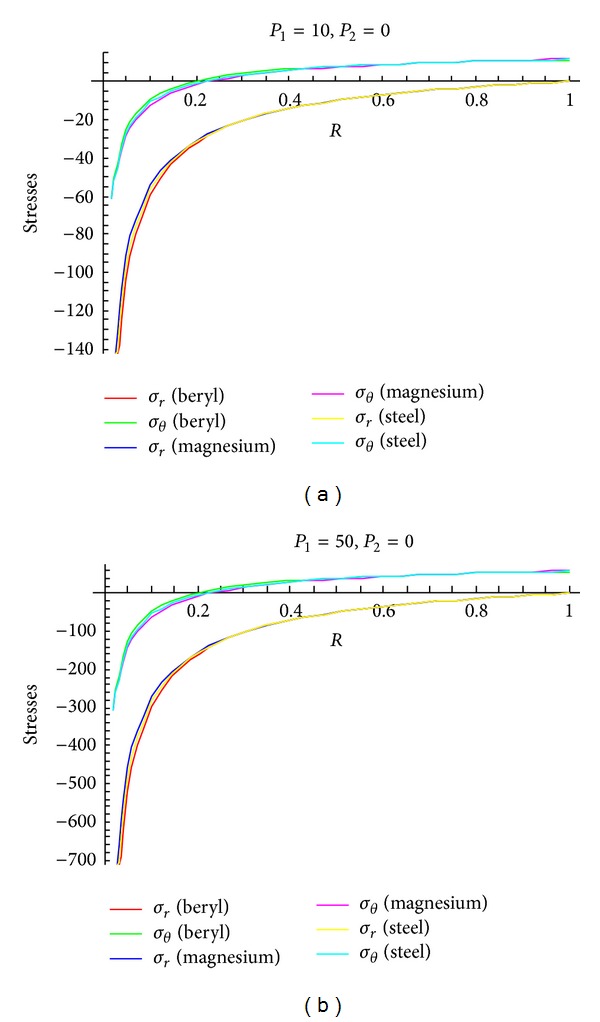
Transitional stresses when internal pressure *P*_1_ = 10 and internal pressure *P*_1_ = 50 (without external pressure).

**Figure 7:**
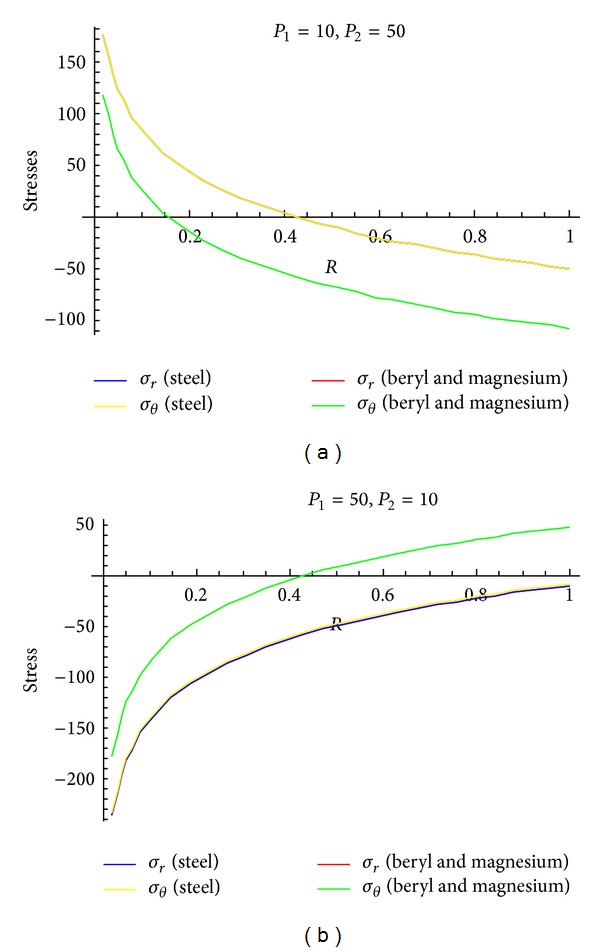
Fully plastic stresses when internal pressure = 10, external pressure = 50 and internal pressure = 50, external pressure = 10, respectively.

**Figure 8:**
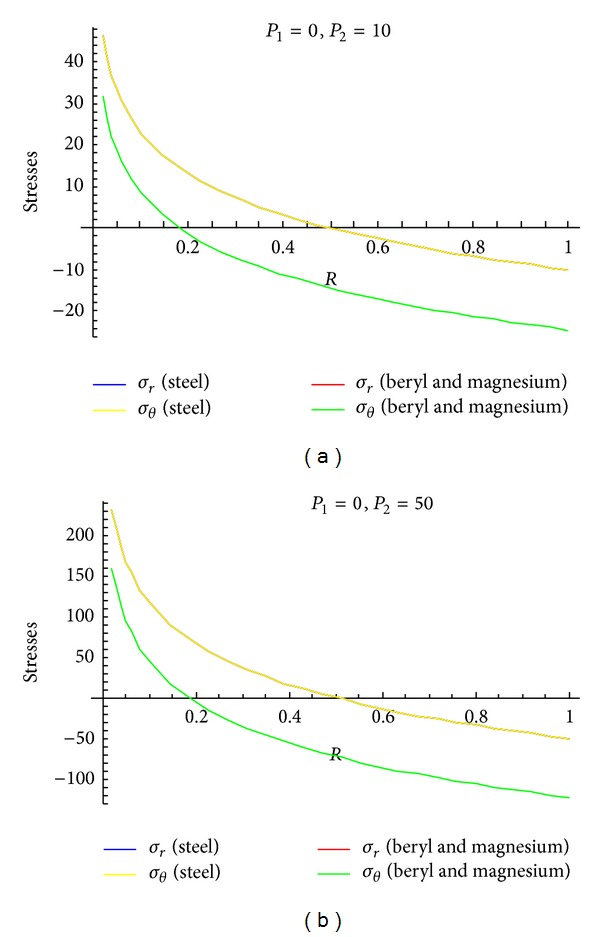
Fully plastic stresses when external pressure *P*_2_ = 10 and external pressure *P*_2_ = 50 (without internal pressure).

**Figure 9:**
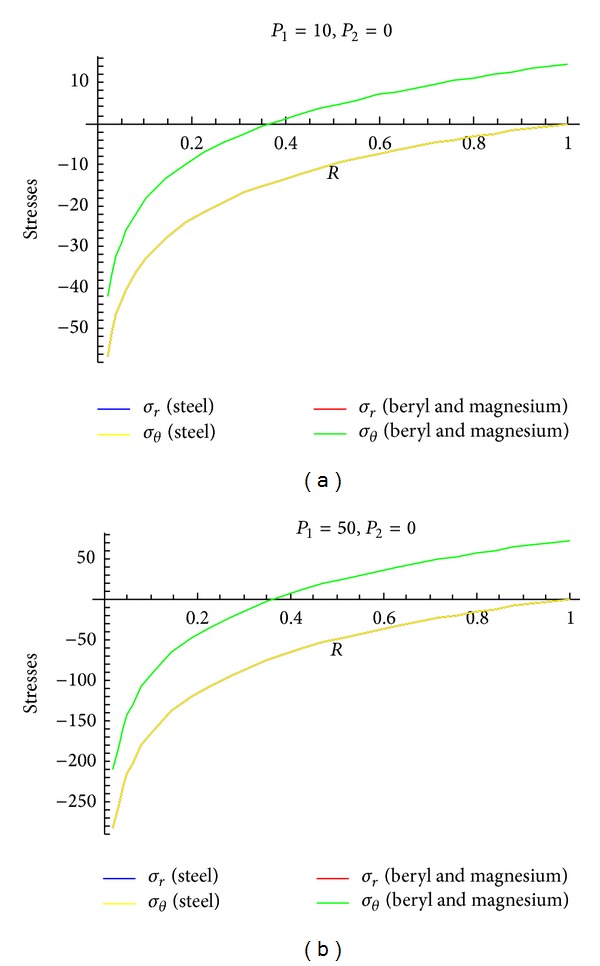
Fully plastic stresses when internal pressure *P*_1_ = 10 and internal pressure *P*_1_ = 50 (without external pressure).

To see the effect of pressure on stresses, Figures 4–6 are drawn for transitional stresses, while Figures 7–9 are for fully plastic stresses.

From Table 5 and Figure 4, it can be seen that stresses are maximum at internal surface. Also it has been observed that the cylinder in which internal pressure is less (compared to external pressure), circumferential stress is less for beryl than that of magnesium and steel. It has been seen from Figure 5 that in case of cylinder having internal pressure more than that of external pressure, circumferential stresses are compressible in nature and maximum at internal surface. Also, it has been noted that, for such type of cylinders, circumferential stresses are less for beryl as compared to magnesium and steel. It can be observed from Figure 6 that transitional stresses are maximum at internal surface for the cylinder under external pressure only.

From Table 5 and Figure 7, it can be seen that fully plastic circumferential stresses are maximum at internal surface for isotropic material as well as for transversely isotropic material. Also, these stresses are high for steel as compared to beryl and magnesium for the cylinder having high external pressure as compared to internal pressure. It has also been noted that stresses are approaching to compressive from tensile for the above cases. Circumferential stresses are compressive in nature for the cylinder whose internal pressure is more than that of external pressure and these stresses approaching towards tensile from compressive as can be seen from Figure 7. It has been observed from Table 5 and Figures 8 and 9 that fully plastic circumferential stresses are again maximum at the internal surface and approaching towards compressive from tensile. Also, these stresses are high for steel as compared to beryl and magnesium. It has also been observed that fully plastic stresses are less for the cylinder under external pressure only as compared to other cases of full plasticity.

## 6. Conclusion

From the above analysis, it can be concluded that circular cylinder under internal and external pressure made up of transversely isotropic material (beryl) is on the safer side of the design as compared to the cylinder made up of isotropic material (steel) as well as of transversely isotropic material (magnesium). The main reason is that the percentage increase in effective pressure required for initial yielding to become fully plastic is high for beryl as compared to steel and magnesium which leads to the idea of “stress saving” that reduces the possibility of collapse of thick-walled cylinder due to internal and external pressure.

## Figures and Tables

**Table 1:** Elastic constants *C*_*ij*_ used (in units of 10^10^ N/m^2^).

Materials	*C*_11_	*C*_12_	*C*_13_	*C*_33_	*C*_44_
Steel (isotropic material)	2.908	1.27	1.27	2.908	0.819
Magnesium (transversely isotropic material)	5.97	2.62	2.17	6.17	1.64
Beryl (transversely isotropic material)	2.746	0.98	0.67	4.69	0.883

**Table 2:** The pressure required for initial yielding and fully plastic state for isotropic material (steel).

Steel (isotropic material)									
Pressure	*P*_*i*_/*P*_*f*_	*a*/*b* = 0.2	*a*/*b* = 0.3	*a*/*b* = 0.4	*a*/*b* = 0.5	((*P*_*f*_ − *P*_*i*_)/*P*_*f*_)∗100			
						*a*/*b* = 0.2	*a*/*b* = 0.3	*a*/*b* = 0.4	*a*/*b* = 0.5
Effective pressure	*P*_*i*_	1.00604	0.84032	0.69384	0.560183	59.9777	43.2757	32.0616	23.7358
	*P*_*f*_	1.60944	1.20397	0.91629	0.693147				

Internal pressure = 0	*P*_*i*_	1.00604	0.84032	0.69384	0.560183	59.9777	43.2757	32.0616	23.7358
	*P*_*f*_	1.60944	1.20397	0.91629	0.693147				

Internal pressure = 10	*P*_*i*_	8.99396	9.15968	9.30616	9.43982	6.70895	3.97012	2.39035	1.40861
	*P*_*f*_	8.39056	8.79603	9.08371	9.30685				

Internal pressure = 50	*P*_*i*_	48.994	49.1597	49.6062	49.4398	1.23158	0.73983	0.45126	0.26881
	*P*_*f*_	48.3906	48.796	49.0837	49.3069				

**Table 3:** The pressure required for initial yielding and fully plastic state for transversely isotropic material (beryl).

Beryl (transversely isotropic material)									
Pressure	*P*_*i*_/*P*_*f*_	*a*/*b* = 0.2	*a*/*b* = 0.3	*a*/*b* = 0.4	*a*/*b* = 0.5	((*P*_*f*_ − *P*_*i*_)/*P*_*f*_)∗100			
						*a*/*b* = 0.2	*a*/*b* = 0.3	*a*/*b* = 0.4	*a*/*b* = 0.5
Effective pressure	*P*_*i*_	1.00261	0.83806	0.69237	0.559264	60.525	43.6614	32.3412	23.9391
	*P*_*f*_	1.60944	1.20397	0.91629	0.693147				

Internal pressure = 0	*P*_*i*_	1.00261	0.83806	0.69237	0.559264	60.525	43.6614	32.3412	23.9391
	*P*_*f*_	1.60944	1.20397	0.91629	0.693147				

Internal pressure = 10	*P*_*i*_	8.99739	9.16194	9.30763	9.44074	6.74451	3.9938	2.40577	1.41822
	*P*_*f*_	8.39056	8.79603	9.08371	9.30685				

Internal pressure = 50	*P*_*i*_	48.9974	49.1619	49.3076	49.4407	1.23843	0.74428	0.45409	0.27063
	*P*_*f*_	48.3906	48.796	49.0837	49.3069				

**Table 4:** The pressure required for initial yielding and fully plastic state for transversely isotropic material (magnesium).

Magnesium (transversely isotropic material)									
Pressure	*P*_*i*_/*P*_*f*_	*a*/*b* = 0.2	*a*/*b* = 0.3	*a*/*b* = 0.4	*a*/*b* = 0.5	((*P*_*f*_ − *P*_*i*_)/*P*_*f*_)∗100			
						*a*/*b* = 0.2	*a*/*b* = 0.3	*a*/*b* = 0.4	*a*/*b* = 0.5
Effective pressure	*P*_*i*_	1.0598	0.87527	0.7164	0.574248	51.8626	37.5544	27.9018	20.7052
	*P*_*f*_	1.60944	1.20397	0.91629	0.693147				

Internal pressure = 0	*P*_*i*_	1.0598	0.87527	0.7164	0.574248	51.8626	37.5544	27.9018	20.7052
	*P*_*f*_	1.60944	1.20397	0.91629	0.693147				

Internal pressure = 10	*P*_*i*_	8.9402	9.12473	9.2836	9.42575	6.14796	3.6023	2.15315	1.26144
	*P*_*f*_	8.39056	8.79603	9.08371	9.30685				

Internal pressure = 50	*P*_*i*_	48.9402	49.1247	49.2836	49.4258	1.123	0.66911	0.40561	0.24056
	*P*_*f*_	48.3906	48.796	49.0837	49.3069				

Where *P*_*i*_ and *P*_*f*_ are pressures required for initial yielding, fully plastic state, while *a* and *b* are internal and external radius of thick-walled circular cylinder.

**Table 5:** Circumferential stresses for different pressures for isotropic (steel) and transversely isotropic material (beryl and steel).

	*R*_0_	Transitional circumferential stresses			Fully plastic circumferential stresses		
		Steel	Beryl	Magnesium	Steel	Beryl	Magnesium
*P*_1_ = 10, *P*_2_ = 50	0	193.946	193.358	197.498	175.754	118.046	118.046
	0.5	−81.4052	−81.5226	−79.6563	−10	−67.7078	−67.7078
	1	−95.7177	−95.6331	−97.0019	−49.42	−107.128	−107.128

*P*_1_ = 50, *P*_2_ = 10	0	−253.946	−253.358	−257.498	−235.754	−178.046	−178.046
	0.5	21.4052	21.5226	19.6563	−50	7.7078	7.7078
	1	35.7177	35.6331	37.0019	−10.58	47.1278	47.1278

*P*_1_ = 0, *P*_2_ = 10	0	50.9865	50.8395	51.8744	46.4386	32.0116	32.0116
	0.5	−17.8513	−17.8807	−17.4141	0	−14.427	−14.427
	1	−21.4294	−21.4083	−21.7505	−9.855	−24.282	−24.282

Where *P*_1_ and *P*_2_ are internal and external pressures.

## Nomenclature

*a* and *b*: Internal and external radii of cylinder
*d*: Constant
*x*, *y*, *z*: Cartesian coordinates
*r*, *θ*, *z*: Cylindrical polar coordinates
*e*_*ij*_ and *T*_*ij*_: Strain and stress tensor
*C*_*ij*_: Material constants
*σ*_*r*_ = (*T*_*rr*_/*C*_66_): Radial stress
*σ*_*θ*_ = (*T*_*θθ*_/*C*_66_): Circumferential stress
*u*, *v*, *w*: Displacement components
*R*: Radial distance
*e*_*ii*_: First strain invariant
*β*: Function of *r* only
*P*: Function of *β* only
*λ* and *μ*: Lame's constants
*R* = (*r*/*b*), *R*_0_ = (*a*/*b*)
*σ*_*z*_ = (*T*_*zz*_/*C*_66_): Axial stress.
